# Exploring Complexity in Out-of-Hospital Clinical Supervision Using Rich Pictures

**DOI:** 10.5334/pme.1674

**Published:** 2025-11-12

**Authors:** Florence M. den Boer, Nelleke Noeverman-Poel, Esther Helmich, Martine C. de Bruijne, Nynke van Dijk, Marianne Mak-van der Vossen, Martin Smalbrugge

**Affiliations:** 1 Amsterdam UMC, Department of General Practice, The Netherlands; 2 Vrije Universiteit, Amsterdam, The Netherlands; 3 Mental Healthcare Institute, GGZ Centraal, Wezep, The Netherlands; 4 Amsta, location Dr. Sarphatihuis, Amsterdam, The Netherlands; 5 Amsterdam UMC, Vrije Universiteit, Department of Public and Occupational Health and Amsterdam Public Health research institute, Amsterdam, The Netherlands; 6 Education and Training, Amsterdam UMC, University of Amsterdam, Department of General Practice and Amsterdam University of Applied Sciences, Faculty of Health, Sports and Physical Activity, Centre of Expertise Urban Vitality, Amsterdam, The Netherlands; 7 Medical Education, Amsterdam UMC, University of Amsterdam, Department of General Practice and Amsterdam Public Health research institute, Quality of Care, Amsterdam, The Netherlands; 8 Amsterdam UMC, Vrije Universiteit, Department of Medicine for Older People and Amsterdam Public Health Research Institute, Aging & Later Life, Amsterdam, The Netherlands

## Abstract

**Introduction:**

The rapidly evolving healthcare landscape in which physicians work and learn is becoming increasingly complex. This growing complexity presents challenges for supervisors and learners, who must balance autonomy with ensuring patient safety. The authors investigated how Elderly Care Medicine and General Practice trainees and supervisors perceive complexity in out-of-hospital settings. The results could contribute to improving the learning and supervision of trainees in complex situations.

**Method:**

From a constructivist paradigm, the authors applied “Rich Pictures” to explore participants’ experiences. Via training institutions, participants were purposefully sampled until data were sufficient to answer the research question. Data collection took place through drawing of supervised complex care situations, directly followed by semi-structured interviews. The authors conducted a reflexive thematic analysis. They analyzed data iteratively, both individually and in “Gallery walks”. Through consultation and discussion, consensus was reached.

**Results:**

Participants described complexity as an intricate interplay of multiple problems in multiple dimensions, where the network of various systems and stakeholders surrounding patients and their interactions and relationships have a major influence on how complexity is perceived. Human interaction was a significant contributor here. For trainees and supervisors, factors related to their working experience and the trainee-supervisor relationship could make a situation more complex.

**Discussion:**

While literature in the field of medical education on complexity mainly describes medical, psychosocial and *intra*systemic elements, our findings indicate that *inter*systemic factors also contribute considerably to how trainees and supervisors in out-of-hospital settings perceive complexity. Recognizing this is an important first step to advance training in the workplace in out-of-hospital settings.

## Introduction

Despite widespread attention to complexity in medical education research [1], little is known about complexity in workplace learning in out-of-hospital settings, such as in General Practice and Elderly Care Medicine. With the insights of this study, we strive to contribute to the understanding of learning and supervising in out-of-hospital residency training, which will benefit teachers and learners in these settings.

Developments and changes in the medical field succeed one another at an ever-growing rate. Constant technological advancements contribute to patients being more connected and informed, resulting in higher expectations of healthcare and receiving more care than in the past [2,3,4], often from many different sources or providers. For example, patients consult online sources about their conditions, which can sometimes lead them to demand specific treatments. Patients live longer, not seldom with multiple conditions [2,5,6]. Doctors must navigate this complex daily practice and so trainees need to be guided in dealing with these complexities.

In medical education literature, complexity principles are approached and described in various ways [1,7,8]. A recent paper by Fustolo et al. builds on a definition by Ladyman et al.: ‘A complex system is an ensemble of many elements which are interacting in a disordered way, resulting in robust organization and memory’ [9], proposing four essential conditions for complexity to arise, namely numerosity, disorder and diversity, feedback and non-equilibrium [10]. Bleakly and Cleland argue complexity shouldn’t be regarded as a research method but rather as a lens. They view complexity as ‘an “extreme” form of non-linearity where things do not always respond the same way to the same “input” depending on other factors, such as context’ [11]. This experience of context-dependency to complexity was also found when surgeons’ perspectives and responses in complex and challenging situations were investigated [12].

A study of data from the US National Hospital Ambulatory Medical Care Survey showed that among 14 different medical specialties, primary care was the most complex of ambulatory settings, it more often concerns the managing of multiple (chronic) problems and accompanying polypharmacy, meanwhile considering different guidelines and coordinating care with various other healthcare professionals [13]. Also, complexity is associated with uncertainty and ambiguity [14]. And while this often can be reduced in hospital settings –for example through diagnostic testing– such an approach is not always feasible or desirable in out-of-hospital settings, for instance due to limited availability of resources [15] or because patients tend to present earlier in the course of their condition. While complexity in out-of-hospital settings, particularly in primary care, has been studied in relation to patient factors, such as multimorbidity or socio-economic status [16,17,18,19,20] there is a notable gap in research addressing its implications for medical education.

Reflecting on medical education, Fraser and Greenhalgh discuss that today’s complexities in healthcare require that ‘we must not merely educate for competence (knowledge, skills and attitude), but for capability (the ability to adapt to change, generate new knowledge, and continuously improve performance)’ [21]. A systematic review on the current position of complexity in medical education found it to be used mainly as a theoretical framework for studying various elements within medical education, such as professionalism, leadership and curricular development [7]. A gap remains in understanding its context and application in day-to-day care of doctors, both trainees and supervisors, in out-of-hospital settings. Here, doctors are at the forefront of patient care and are often the principal care providers for patients [15]. They are expected to oversee the ever-changing healthcare landscape and face unique challenges in providing holistic, adaptive care to their patients [22].

In this study we aim to first gain insight in the practical meaning of complexity for workplace learning of trainees and their supervisors in out-of-hospital settings. Understanding this could be a starting point toward pragmatic recommendations for learning and supervision in these settings. Therefore, we set out to investigate the following research question: How do Elderly Care Medicine and General Practice trainees and supervisors perceive complexity in workplace learning in out-of-hospital settings?

## Method

We conducted a qualitative study to investigate how trainees and supervisors in out-of-hospital settings perceive complexity in clinical practice. We used inductive, reflexive thematic analysis [23]. From a constructivist paradigm, in which knowledge is seen as actively constructed as a result of human interactions and relationships [24,25], we chose Rich Pictures (RPs) to explore experiences of trainees and supervisors [26]. This free-form drawing method is derived from Systems Engineering and helps explore complex situations by depicting all important aspects of a situation [27]. It is particularly suited to elicit tacit information and holistically capture the multiple interactions and dimensions of situations from the participant’s perspective [26,28]. Therefore, this method is well-placed to contribute to the exploration of complexity in our study. Data was collected through drawing of RPs of complex care situations, directly followed by individual semi-structured interviews.

### Participants

We included trainees and supervisors from three General Practice (GP) and Elderly Care Medicine (ECM) training institutes at academic medical centers across the Netherlands, i.e. Radboudumc and Amsterdam University Medical Centers’ locations VUmc and AMC. GP and ECM are major primary care disciplines in the Netherlands, characterized by a generalist, holistic and person-oriented approach to patient care [29,30]. General Practitioners (GPs) provide primary care and are gatekeepers to hospital-based care [31]. Elderly Care Physicians (ECPs) mainly provide care in nursing homes and for community-dwelling elderly people in collaboration with GPs [30,32].

Participants were recruited by email, via the secretaries of the residency training programs, by giving short oral introductions on teaching days and through the researchers’ networks. We included participants of different age and gender, and with various working experience. We purposefully sampled until theoretical sufficiency was reached: the point where we had sufficient data to describe and explain the relevant topics and themes without gaps or leaps of logic [33,34].

### Data collection

Participants were asked beforehand by email to think of complex cases that they had experienced and to reflect on the meaning of the word ‘complexity’. They were not provided with a definition as we were looking for their personal experience of complexity. Each interview began with instructions about RPs, using the guidelines as described by Armson [35]. Participants were then asked to draw RPs and were subsequently interviewed. The interview guide is available as Supplemental Digital Appendix 1.

Participants could choose from sheets of paper (sizes A0 and A3) and pencils in several colors. The interviewer left the room while the participant was drawing, which usually took 20–30 minutes. Trainees were asked to draw two complex situations: one in which they received a lot of supervision and one which they handled more or less autonomously. Initially, supervisors were also asked to draw both types of situations, related to them giving supervision. However, as it appeared difficult for them to draw situations in which the trainee worked rather autonomously, we decided to only ask them to draw a complex situation in which they were closely involved as supervisors.

### Data analysis

Data analysis included a combination of iconographic analysis of the RPs [26,28] and reflexive thematic analysis of the interviews based on inductive coding [23], which was carried out in an iterative way. Analysis started during the interview, when NN together with the participant began the iconographic analysis and tried to make sense of elements in the RPs, their features such as size, color, proportions; their meaning to the participant and discussed relationships between them [26]. After every interview, NN immediately logged a first reflection. Interviews were then transcribed verbatim. Analysis of both RPs and interviews took place in two subsequent rounds, due to a change in the research team. Data collection was completed by the initial research team, which also carried out the first round of analysis. Following a break of approximately two years –caused by personal circumstances– a second team, with a slightly different composition, undertook a second round of data analysis. This had a twofold objective. Firstly, we wanted to verify that the previous analysis still held relevance and resonated after the time gap. Secondly, we took this as an opportunity, to broaden and deepen our insights and actively look for additional perspectives.

The first round of data analysis was led by NN and EH and started with open coding of the transcripts, leading to the identification of initial codes. Other team members were involved in viewing sessions, in which the research team discussed the first five RPs accompanied by their respective interviews with the same approach as during the interviews and identified visual motifs and icons, exploring their possible meanings. Input from the viewing sessions and gallery walks (see below) was used for focused coding of the interview transcripts. Codes were combined into categories and broader themes.

Gallery walks are suited to analyse the whole set of RPs, specifically looking for similarities, differences and intrinsic connections throughout the complete set [28]. All RPs were displayed in random order on the wall of a room. Contributors of the gallery walk individually crossed the room to examine the RPs for 30 min, followed by a 1-hour group discussion about motifs and themes, which was audio recorded and summarized in a report. The first gallery walk was performed with all researchers involved in the project (NN, MB, ND, MS, EH) supported by an RP expert, Sayra Cristancho. The second gallery walk was carried out by part of the research team (NN, EH, MS) complemented with researchers familiar with RPs but outsiders to GP and ECM residency training, allowing for unanticipated perspectives on the data.

After a change in the research team, a second round of data analysis was carried out by FB and aimed to review and enhance the understanding of the coding first done by NN and EH. The thematic analysis was continued, further developing the previously identified categories and themes and examining relationships among themes. Two additional gallery walks were performed to determine whether the subject and themes were still viewed as relevant to doctors in the field we were researching and held true for them, as two years had passed between the first and second round of data analysis. The gallery walks were carried out in the same manner as described above. First, we discussed the RPs with part of the research team (FB, EH, NB, MM) and members of a GP research group, which also included practicing GPs and trainees. Then a final gallery walk was carried out by FB, EH and MM together with residents in training of both specialties at a postgraduate training facility. We have ensured a diverse group participation in the gallery walks, which aided us to engage in reflexivity, critically examining our own thoughts and reasoning through hearing from the multiple perspectives. This experience added to the richness of the interpretation, refining and expanding it. Motifs and themes identified were discussed with the whole research team to structure a coherent narrative and final outline of themes.

### Research team

FB is a PhD candidate and GP in training, NN is an Elderly Care Physician (during the period of data collection she was still in training); both received training in interview techniques, visual methods and qualitative research. As a ECP in training NN could relate well to the day-to-day work of the participants and could discuss their cases on a more profound level. Having FB as a GP in training further analyse the data, added to a more comprehensive total perspective as participants from both specialties were included in this research. The research team also included experts in visual methods and complexity theory (EH), medical education research (ND, EH, MM), patient safety (MB), General Practice (ND, MM), and Elderly Care Medicine (MS, EH). Through our personal experiences with the complexities of working in out-of-hospital care (FB, NN, MP, EH, MM, MS) and education in both General Practice (ND, MM) and Elderly Care Medicine (MS, EH) we ensured a holistic and reflexive approach. The team was able to relate to the subject with a broad scope, while the presence of relative outsiders to primary care workplace learning (MB) allowed for a critical view on the insiders’ perspectives. Throughout the process, we maintained an ongoing dialogue regarding the data, developing insights, and their interpretation. We carefully considered the change in the main researcher (from NN to FB). FB was given sufficient time to thoroughly engage with the subject and provided a critical re-examination, while NN remained involved, ensuring a complete transfer of all data. We believe the variety in authors and their experience provided for a thorough analysis of the data.

### Ethical considerations

The study was approved by the ethical review board of the Netherlands Association for Medical Education (NVMO), file number 591. All participants provided written informed consent. No incentives were provided for participation. Participant data was anonymized and stored anonymously.

## Results

We conducted 17 interviews, with 8 trainees and 9 supervisors. Table 1 shows the general characteristics of the participants. Participants drew a total of 26 RPs, representing a total of 23 unique patient cases. Participants are hereafter referred to by their specialty (ECM or GP), T for trainees and S for supervisors, and a unique number (e.g. ECM-T2 for ECM trainee number 2).

**Table 1:** Participant characteristics.


TRAINEES				

	AGE (YEARS)	GENDER	YEAR OF RESIDENCY TRAINING	EXPERIENCE AS PHYSICIAN BEFORE ENTERING RESIDENCY TRAINING (YEARS)

ECM-T1	30	Female	1^st^	3

ECM-T2	31	Female	3^rd^	5

ECM-T3	27	Female	3^rd^	0.8

ECM-T4	26	Female	1^st^	1.3

ECM-T5	36	Male	2^nd^	2.6

ECM-T6	40	Female	1^st^	14

GP-T1	29	Female	3^rd^	2.5

GP-T2	30	Female	1^st^	1.9

**SUPERVISORS**				

	**AGE (YEARS)**	**GENDER**	**EXPERIENCE IN ECM/GP (YEARS)**	**EXPERIENCE AS SUPERVISOR (NO. RESIDENTS SUPERVISED)**

ECM-S1	47	Female	19	3

ECM-S2	44	Female	12	3

ECM-S3	51	Male	8	3

ECM-S4	58	Male	>25	15

ECM-S5	50	Female	17	5

ECM-S6	50	Female	12	4

GP-S1	44	Male	11	3

GP-S2	39	Female	7	2

GP-S3	55	Male	26	17


ECM = Elderly Care Medicine GP = General Practice T = Trainee S = Supervisor

We identified four themes that contributed on a macro, meso, and micro level to how GP and ECM trainees and supervisors perceive complexity: intersystemic factors, human interaction, work experience and trainee-supervisor relationship (see Table 2).

**Table 2:** Themes.


THEMES RELATED TO COMPLEXITY	LEVEL	CHARACTERISTICS	EXAMPLES OF IMAGES IN RICH PICTURES

*Intersystemic factors*	Macro	Multiple stakeholders across different systems (e.g. hospital care, mental care, home care etc.), their relations and reciprocal influence, transitions	A multitude of (stick) figures depicting patients, family, healthcare professionals, arrows, vehicles, buildings, institutions

*Human interaction*	Meso	Multiple interests and perspectives, diversity, emotions, communication	Telephones, computers, depicted emotions, question marks, thinking and talking balloons, symbols e.g. crosses and lightning

*Work experience*	Micro	Medical complexity, multimorbidity, time pressure	Internal organs, medication, syringes, medical devices, clocks, calendars
	
*Trainee-supervisor relationship*		Coaching of trainees by supervisors, trainees receiving guidance from supervisors	(Stick) figures depicting trainee and supervisor


### Intersystemic factors

**Figure 1:**
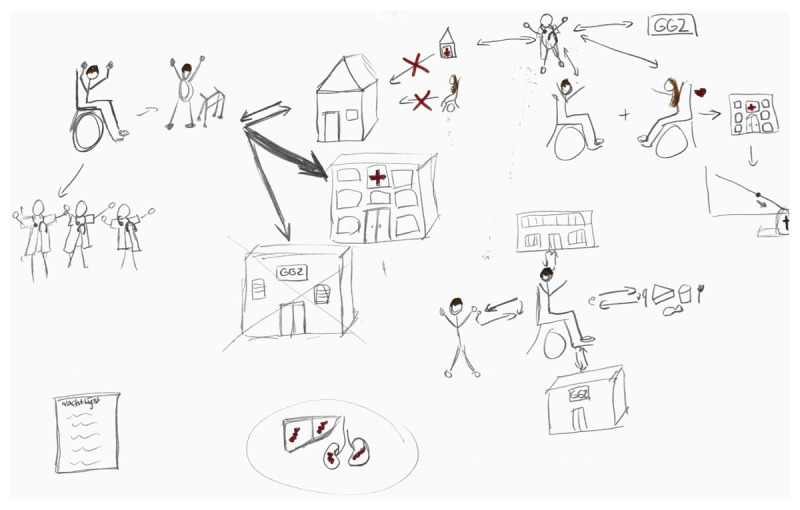
RP by ECM-T4 depicting the patient in different systems and different interactions between them.

The most outstanding features of the RPs were buildings, names of institutions, vehicles, telephones, informal and formal caregivers, and arrows that indicated interactions and transitions between various care systems (see Figure 1). These features appeared consistently throughout the dataset (22 out of 26 RPs). The visual motifs and accompanying interview transcripts indicated that most participants identified the involvement of many different systems (such as multiple healthcare institutions, different individual care providers and informal caregivers) in the care for those patients on a macro level:

‘What I have drawn here is that there are mainly many doctors involved with that patient. Uhm, patient goes to the hospital very often, does and doesn’t go home. So he has been home a few times, admitted to the institution again and then home again. Went to the Mental Health Service. However, the patient no longer wants to go there at the moment, which is why there is drawn a cross. And what you see here is that… uhm he came in a wheelchair and has actually improved so much that the doctors think: “Hey, you can go home again.” This is the GP, with a GP sign next to it. That’s the GP who doesn’t think that’s possible. The partner actually does not want the husband to go home.’ (ECM-T4)

From our data the participants described complexity as an intricate interplay of multiple problems in various patient-related dimensions, where the network of the various systems and stakeholders surrounding patients on a macro level and their interaction and relationship with each other had a major influence. We labelled this intersystem complexity.

‘It is not only the patient who is complex, but also the entire network around it that can make it sometimes rather complex.’ (GP-S1)

### Human interaction

**Figure 2:**
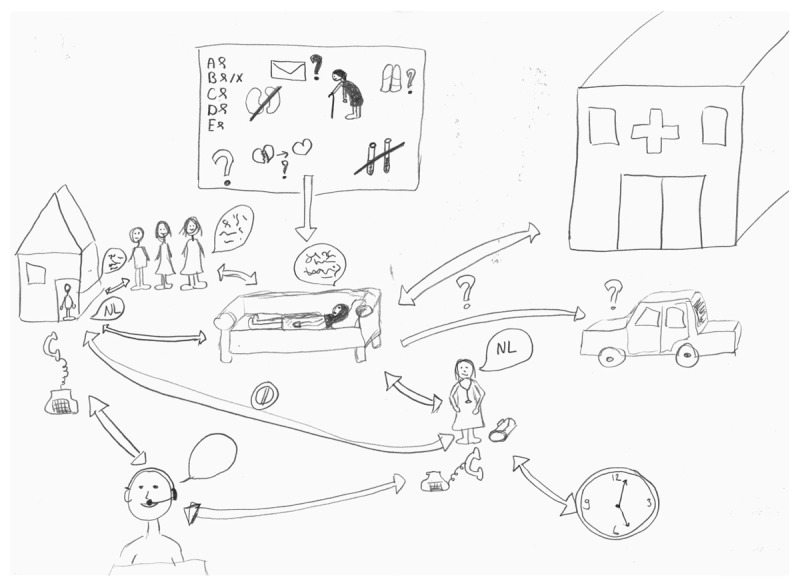
RP by GP-T1 with different languages depicted in text balloons.

Participants closely associated complexity with human interaction and communication, characterized by multiple interests, emotions, cultural diversity and language barriers (see Figure 2). This interaction was particularly challenging when multiple parties communicated simultaneously about a case:

‘Many changes in healthcare providers, where the communication between, uhm yes, certain things that were passed on from the hospital to the healthcare providers did not get through to me. Certain information that family provided to the nursing staff also did not reach me.’ (ECM-T4)

‘We had really thought about it carefully. Also with all doctors, with the family, with the psychologist, with experts we’d consulted. So yes, that makes it even more difficult. […] I have concluded for myself what I should learn from this: that there are simply several, truly multiple, views.[…] there are different interests at stake here that make it complex. There are a lot of catches.’ (ECM-T6)

Emotions played a significant role in shaping communication. In 22 of the 26 RPs of complex situations participants depicted one or more emotions, this was further supported by the data from the interview transcripts.

‘The daughter with the bun, that was really… well, if looks could kill, I wouldn’t be having this conversation with you here. It was completely negative, but it also contained its own sadness, unresolved…’ (ECM-S1)

Multiple interests and perspectives were often described as adding to complexity and could cause disagreements, but could sometimes also bring about practical solutions:

‘She [informal care giver] is actually always present at those conversations, and sometimes when he [the patient] walks in and we wonder; “What is really going on?” we call her and she can tell us what is really going on with him, and we often do call her. And that’s nice, yes.’ (GP-T2)

Diversity could influence the complexity of cases. As different cultural backgrounds could raise different expectations of stakeholders, while language barriers could bring about an added challenge in complex situations:

‘I went to the house, I found an old lady on the couch who didn’t speak a word of Dutch. With a very sweet family around her, who looked at me just as questioningly, who didn’t speak a word of Dutch either. So that started well. […] I said “I’m worried about that [the patient’s condition]. Uhm, what should we do now?” Well that might also have been a cultural thing that was said about it: “Yes, no, that’s for the doctor to decide”.’ (GP-T1)

Besides the complicating impact on communication, positive aspects of cultural diversity were also mentioned by participants:

‘What I often see with complex care for migrants […] that there is a very good network in terms of accessibility and informal care. So that’s very nice. Uhm… and sometimes there is more need, even more need for explanation.’ (GP-S2)

Thus, at a meso level, human interaction contributed significantly to perceived complexity.

### Work experience

For trainees and supervisors different factors could make a situation complex. When explaining what complex cases were to them, both trainees and supervisors referred to intersystem complexity and the importance of human interaction, but the difference was found to be related to their work experience. Trainees mostly described cases in which multiple problems in various patient-related dimensions played a role: e.g. multi-morbidity, dependency in activities of daily living, social and psychological problems. Trainees often drew internal organs, medication, syringes and medical devices to depict different diseases and conditions.

‘Something can be complex because it is medically complicated, but also the social situation or uhm… even the intellectual capacity of a patient for example. And what makes it even more complex is that it cannot be treated according to certain guidelines. I think that is complex for me.’ (GP-T1)

Although supervisors also did describe these types of cases as complex, they were able to manage these aspects of complexity and seemed to struggle less with them because of their working experience. Complex cases were at times described by them as ‘business as usual’.

Over a third of the participants drew one or more clocks. Time pressure was mainly expressed by the trainees during the interviews.

‘…then I also think: ‘Oh dear, I actually have to finish this, but I have to go there now too and how am I going to manage that in time?’ And well, here I was again, and you’ll soon be another hour before you’re, uhm, ready to leave again. So you do feel a bit of time pressure. On such a, uhm, on such a day. Yes. That’s that little clock. […] I thought ‘Okay, I have to do this, that and all that, but I also have to go to several locations and how am I going to do all that?’ I find that complex.’ (ECM-T3)

In contrast, supervisors seemed to benefit more from their experience in managing time as none of them discussed time pressure as a contributing factor to complexity.

### Trainee-supervisor relationship

**Figure 3:**
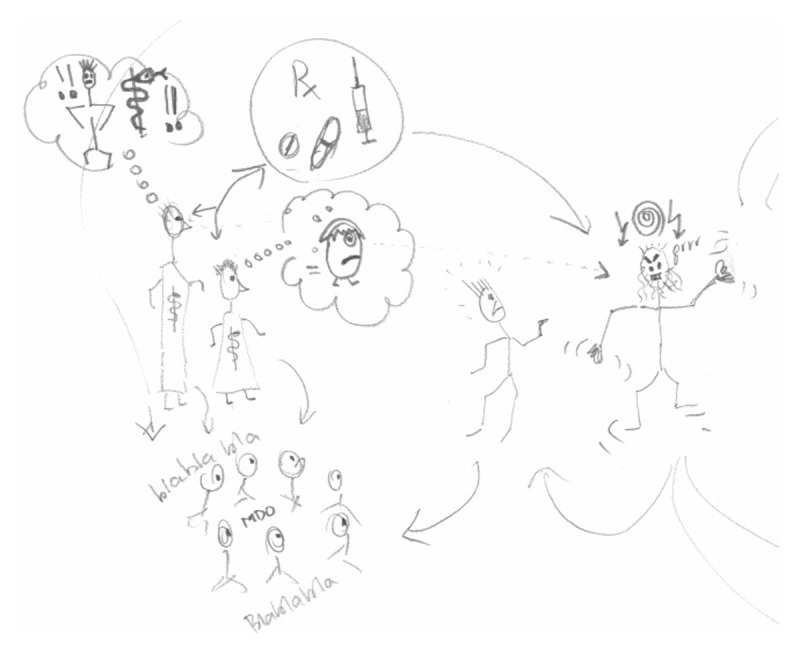
Cutout of RP of supervision situation by ECM-S3.

**Figure 4:**
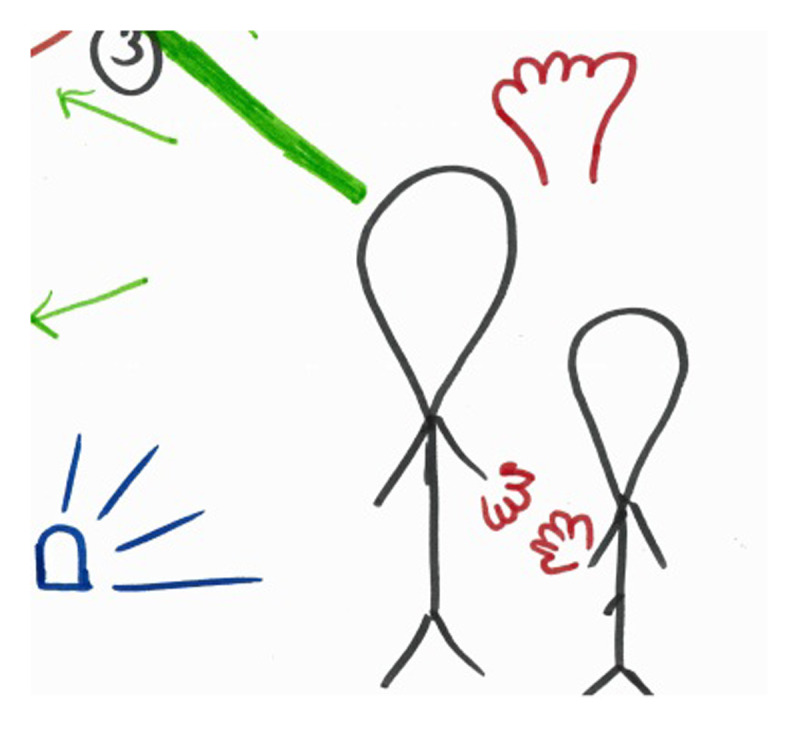
Cutout of RP of trainee-supervisor relationship by ECM-T2.

Both trainees and supervisors explicitly made drawings of the trainee-supervisor relationship (see Figures 3 and 4). Trainees often experienced support from their supervisors which helped them manage complex cases.

‘This is me and my supervisor. I’m the slightly smaller one, right, because I have less experience. And we hold hands, so I get a lot of support from her […] actually from the start, it was of course already a complex case because the man had everything wrong with him, she also supported me in that, oh…, during the debriefing. Like: “with medication try this, that”, “think about that”.’ (ECM-T2)

Supervising the trainee could increase complexity for supervisors because they often had to manage a situation indirectly through their trainee. This added a layer of complexity for supervisors as they had to rely on the trainee’s abilities to handle the situation and communicate effectively with all stakeholders involved.

‘That also means that my involvement in this complex case is more intensive. Because I think that, uhm, with this trainee, he is doing well in terms of medical knowledge, but in terms of positioning, he can be more powerful. And that makes my involvement a little stronger.’ (ECM-S3)

Supervisors explained how their role required them to step back and allow the trainee to take the lead, which could be challenging. This dynamic sometimes made it difficult for supervisors to intervene directly and resolve issues, as they had to guide the trainee to manage the situation themselves. Supervisors had to balance providing support and allowing autonomy. On the other hand, if a trainee was handling a complex situation well, the supervisor could really enjoy taking a backseat.

‘My trainee, a first-year, is still too timid about that. So when a question is asked, it can take ten seconds before an answer comes. […] Then I thought: “Well, we have to supervise this properly and I cannot leave the trainee alone in this.” Precisely because in the field of communication, that is where his learning goals lie: clarity, appearing concrete and powerful, appearing decisive. And I think for that very reason, I thought it would be good to uhm, stand next to him.’ (ECM-S3)

‘I also consciously drew myself a little smaller […] in the end, uhm, I thought this role was actually a delicious one. Like, well I’m going to sit back and literally wait in the sun until she comes with good or bad news. […] A completely different role relationship. Which I clearly feel comfortable with […] I don’t feel any pressure at all. And of course I heard that the nursing assistants were not completely satisfied with that, but then I thought: “Yes, it’ll be fine. She’ll be fine”.’ (ECM-S1)

In conclusion, clinical situations often became more complex for supervisors due to the training context.

## Discussion

Our study aimed to construe how Elderly Care Medicine and General Practice trainees and their supervisors perceive complexity in out-of-hospital settings. We identified four main themes which contribute to how they perceive this complexity: intersystemic factors, human interaction, work experience, and trainee-supervisor relationship. From our data, participants described perceived complexity as an intricate interplay of multiple problems in multiple patient-related dimensions, in which the network of the various systems and stakeholders surrounding patients, and their interactions and relationships had a major influence. On a macro level we considered this to be intersystem complexity. These findings broadly align with complexity theory often used in healthcare [1], where systems are considered as ‘linked components’, you cannot view (parts of) these systems in isolation; their relationships influence each other [36].

The emphasis on intersystem complexity is what our study can contribute to the medical education literature. Many conceptualizations of complexity in out-of-hospital settings are predominantly described with the intrasystemic factors we also encountered in our study, such as medical complexity, multimorbidity, and the dynamics within systems [16,17,18,19,20]. While it is important for trainees to learn to manage complexity at the patient level, intersystem complexity at a macro level must also be considered as an essential component of what influences complexity in out-of-hospital settings.

Recognizing the intersystemic factors of complexity may be challenging for trainees, as it is a learning process and they are relatively unexperienced. As the RP technique elicits additional information through the act of drawing and aids in making implicit feelings and perceptions become apparent [26], we suggest RPs could even be used as an effective tool to visualize and pinpoint where complexities in cases lie. Further, intraprofessional learning might help improve collaboration and interaction of different medical disciplines across systems (e.g. hospital care vs. out-of-hospital care by doctors) as it could provide better insight into other systems and how they function [37]. Effective supervisor guidance could help trainees to bring some order, coordinate, identify where complexity and uncertainty can best be accepted, and where complexity can be reduced [10,15]. Supervisors, through their own working experience and perhaps through their experience with supervision, can support trainees with intersystemic factors, although it requires supervisors to be aware of this macro level of complexity first. We suggest supervisors receive support in this area through their existing educational programs. In addition, it would be interesting to gather their specific opinions on this matter and also to examine the impact of supervisors’ experience with supervision. We acknowledge that this aspect is not currently addressed in our research, yet we believe it could be relevant.

Our study has some strengths and limitations. One potential limitation is that interviewing can lead to socially desirable responses [38]. Therefore, it is possible that supervisors would share less of their doubt and uncertainty with a researcher who is a trainee as well. However, the fact that the interviews were conducted by a peer (to the trainees) or someone lower in the hierarchy (to the supervisors) might have mitigated this effect. Observations can complement and further enrich our data here; this is planned for a future study.

Another possible limitation is that the medical educational structures and organization of healthcare in out-of-hospital settings can vary significantly between countries. In our study we observe a gender distribution among participants, with a generational shift between trainees and supervisors, that aligns with the situation in the Netherlands [39,40,41,42]. We have aimed to generate a diverse sample and believe we achieved a well-balanced mix of participants, which contributes to the richness of the data. Our results may be influenced by the specific context of the Netherlands. However, we believe this has only limited impact and that our findings are likely transferable to situations where there are many different stakeholders and systems involved in patient care.

We ultimately consider the change in research team composition to be a strength of this study. Initial analysis focused primarily on intersystem complexity. Revisiting the data –through fresh perspectives and a partially renewed team– led to new insights, revealing that additional factors such as human interaction, work experience, and the interpersonal trainee-supervisor relationship substantially shape how our participants perceive complexity.

Future research should explore whether and how adequate supervision can support trainees in managing complexity. Our study offers a first step by describing how complexity is perceived by our participants and this may support recognizing complex situations. It is important to further investigate how complexity is recognized and also how it is navigated by trainees and supervisors, as this can inform shaping of curricula.

We conclude that residents and supervisors in out-of-hospital settings experience complexity not only in patient-related and intrasystem factors; they rather extend beyond this in intersystem complexity and the coordination between systems. Recognizing this broader vision on complexity is an important first step to improve training in the workplace in out-of-hospital settings.

## Previous presentations

This study was presented at the NVMO (Netherlands Association for Medical Education) conference, May 2024 and at the AMEE (Association for Medical Education in Europe) conference, August 2019.

## Additional File

The additional file for this article can be found as follows:

10.5334/pme.1674.s1 Supplemental Digital Appendix 1. Interview guide.
