# Supplementary material for: Exploring Complexity in Out-of-Hospital Clinical Supervision Using Rich Pictures
Source: Perspect Med Educ. 2025 Nov 12;14(1):761–72. doi: 10.5334/pme.1674 (PMC12617401; doi:10.5334/pme.1674)
Supplement: Supplemental Digital Appendix 1. — Interview guide. [file pme-14-1-1674-s1.pdf]

Interview guide

1. Start
- Welcome, getting to know each other, explanation about the study
2. Questions
- What do you think of when I say 'complexity'?
- What do you think of when I say 'uncertainty'?
3. Instructions for drawing
- Explanation of Rich Picture method, incl. showing an exemplar Rich Picture
- Explanation of assignment
4. Drawing session (max. 30 min, interviewer leaves the room)
5. Questions after drawing session
- What did you draw? Why did you choose to draw this situation?
- What made the situation complex?
- About which aspects of the situation did you feel certain? What made you feel uncertain?
- Supervision:
  - Residents: When did you ask for supervision? What was the immediate reason for you to ask for supervision? What was the role of your supervisor? How did you and your supervisor decide on the management of the case? What was the difference between case 1 and 2?
  - Supervisors: When and why were you involved in the situation? Which tasks did you entrust to your trainee? Which tasks did you not entrust? What was your role in this case? What did you expect from your trainee? How did you and your trainee decide on the management of the case?
- How did you ensure patient safety in these cases?
